# Sex-Related Differences in Clinical Characteristics and Outcomes Among Patients Clinically Managed for Suspected Bacterial Infection in the Emergency Department

**DOI:** 10.3390/medicina62091776

**Published:** 2026-09-16

**Authors:** Daian-Ionel Popa, Larysa Alexandra Bălulescu, Ovidiu Alexandru Mederle, Codrina Mihaela Levai, Tiberiu Buleu, Anca Tudor, Ion Petre, Raluca Ibănescu, Carmen Gabriela Williams, Dumitru Sutoi, Dragos Fortofoiu, Cătălina Băzăvan, Adelin Ciudoiu, Răzvan Roșca, Florina Buleu

**Affiliations:** 1 Research Center for Medical Communication, “Victor Babeș” University of Medicine and Pharmacy, 300041 Timisoara, Romania; daian-ionel.popa@umft.ro (D.-I.P.); codrinalevai@umft.ro (C.M.L.); 2 Emergency Municipal Clinical Hospital, 300254 Timisoara, Romania; raluca.ibanescu@umft.ro (R.I.); williams.carmen@umft.ro (C.G.W.); catalina.bazavan.umfvbt@gmail.com (C.B.); adelin.ciudoiu@umft.ro (A.C.); 3 Department of Surgery I, Emergency Medicine Discipline, “Victor Babeș” University of Medicine and Pharmacy, 300041 Timisoara, Romania; dumitru.sutoi@umft.ro; 4 Faculty of General Medicine, “Victor Babeș” University of Medicine and Pharmacy, 300041 Timisoara, Romania; tiberiu.buleu@umft.ro; 5 Department of Functional Sciences, “Victor Babeș” University of Medicine and Pharmacy, 300041 Timisoara, Romania; atudor@umft.ro (A.T.); petre.ion@umft.ro (I.P.); 6 Doctoral School, Faculty of General Medicine, “Victor Babeș” University of Medicine and Pharmacy, 300041 Timisoara, Romania; 7 Center of Advanced Research in Cardiology and Hemostaseology, “Victor Babeș” University of Medicine and Pharmacy, 300041 Timisoara, Romania; 8 Doctoral School, University of Medicine and Pharmacy of Craiova, 200349 Craiova, Romania; fortofoiudragos@gmail.com; 9 Clinical Practical Skills Victor Babes, Departamentul I Nursing, 300310 Timisoara, Romania; 10 Department for the Prevention and Control of Healthcare-Associated Infections (CPIAAM), Clinical Hospital of Infectious Diseases and Pulmonology “Dr. Victor Babeș” Timișoara, Gheorghe Adam Street No. 13, 300310 Timisoara, Romania; razvan.rosca@rezident.umft.ro; 11 Cardiology Department, “Victor Babeș” University of Medicine and Pharmacy, 300041 Timisoara, Romania; florina.buleu@umft.ro

**Keywords:** emergency department, infection source, sex, biomarkers, comorbidities, inflammatory biomarkers, suspected infection

## Abstract

*Background and Objectives*: Suspected bacterial infection is one of the leading presentations to the Emergency Department (ED) and is still associated with considerable morbidity and mortality. There is increasing evidence that biological sex may influence host immune responses, disease manifestations, therapeutic requirements, and clinical outcomes in infectious diseases. However, sex-specific differences among patients clinically managed for suspected bacterial infection in ED remain incompletely defined. This study, therefore, aimed to assess demographic characteristics, clinical presentation, and clinical management as well as short-term outcomes related to sex among these patients. *Materials and Methods*: This retrospective single-center observational study included consecutive adults presenting with suspected bacterial infection to the ED between 1 June and 31 August 2025. Demographic, clinical, laboratory, treatment, infection-source, and outcome data were extracted from medical records. Between-group comparisons were followed by planned age-adjusted analyses and multivariable logistic regression. *Results*: A total of 213 patients were included, comprising 100 women (46.9%) and 113 men (53.1%). Women were older than men (median 77.0 vs. 67.0 years, *p* < 0.001), had lower diastolic blood pressure (70 vs. 75 mmHg, *p* = 0.004), and lower hemoglobin concentrations (11.79 vs. 12.45 g/dL, *p* = 0.004). Cardiovascular disease was more frequent among women than men (72.0% vs. 52.2%, *p* = 0.003), although this difference was attenuated and no longer significant after adjustment for age. A documented urinary source of infection was more frequent among women (51.0% vs. 34.5%, *p* = 0.015) and remained significantly associated with female sex after age adjustment (OR 2.14, 95% CI 1.20–3.81, *p* = 0.010; Benjamini–Hochberg-adjusted *p* = 0.039). No significant difference was detected in Emergency Department treatment: supplemental oxygen, vasopressors and broad-spectrum antibiotics were given with similar frequency to women and men (all *p* ≥ 0.139). Direct intensive care unit (ICU) admission from the ED occurred in 14 of 100 women (14.0%) and 21 of 113 men (18.6%; OR 0.71, 95% CI 0.34–1.49), and in-hospital mortality in 7 women (7.0%) and 9 men (8.0%; OR 0.87, 95% CI 0.31–2.43). After adjustment, female sex was not independently associated with direct ICU admission (OR 0.64, 95% CI 0.29–1.39; *p* = 0.257) or in-hospital mortality (OR 0.80, 95% CI 0.27–2.35; *p* = 0.690). *Conclusions*: In this single-center retrospective cohort of adults clinically managed for suspected bacterial infection in the emergency department, women were older and more frequently had a documented urinary source of infection. After adjustment for age, female sex remained associated with a documented urinary source of infection. The study did not provide precise evidence that sex was independently associated with direct ICU admission or in-hospital mortality; however, the confidence intervals were wide and clinically relevant differences cannot be excluded. Larger prospective multicenter studies with standardized diagnostic criteria, microbiological confirmation, and adequate outcome-event numbers are needed.

## 1. Introduction

Suspected bacterial infection represents one of the most common causes of presentation to the Emergency Department (ED) and remains a major contributor to morbidity and mortality worldwide [1]. Early recognition and timely initiation of appropriate treatment are essential in preventing progression toward severe infection, sepsis, or septic shock. Because infection presentation in the ED is often heterogeneous and nonspecific, emergency physicians frequently rely on a combination of clinical assessment, vital signs, inflammatory biomarkers, and imaging studies to rapidly identify patients at risk of deterioration [2]. Delayed diagnosis and treatment have been associated with prolonged hospitalization, increased need for intensive care, and higher mortality rates [3,4,5].

In recent years, attention has increased on how biological sex may influence the host response to infection. Sex-related differences have been described in immune system activation, inflammatory response, hormonal regulation, and susceptibility to infectious diseases [6]. Experimental and clinical studies suggest that female patients may exhibit stronger innate and adaptive immune responses than males, potentially leading to differences in clinical presentation, severity, and outcomes during infectious processes [7,8,9].

Several studies have reported that male patients are more likely to develop respiratory infections and bacteremia, whereas urinary tract infections are more frequently encountered in female patients [10,11] or showed similar clinical characteristics and short- and medium-term mortality with females, despite a lower grade of organ dysfunction among women [12]. In addition, differences in comorbidities, cardiovascular status, inflammatory biomarkers, and patterns of organ dysfunction may contribute to variations in treatment requirements and prognosis between the sexes [8,9,13]. Despite these observations, the impact of sex on outcomes among patients clinically managed for suspected bacterial infection in the emergency department remains incompletely understood, and existing literature has produced conflicting results regarding mortality, intensive care admission, and disease severity [13,14,15].

The Emergency Department represents a unique clinical environment in which rapid decision-making is required before microbiological confirmation becomes available [1]. Consequently, studying patients clinically managed for suspected bacterial infection in the emergency department, rather than only those with confirmed sepsis, may provide a more realistic overview of the early stages of infection management in emergency care. Identifying sex-related differences in this population could improve risk stratification, support individualized treatment approaches, and optimize emergency department protocols.

Therefore, this study aimed to evaluate sex-related differences in demographic characteristics, comorbidities, laboratory findings, infection sources, therapeutic interventions, and short-term outcomes among adult patients presenting to the Emergency Department with suspected infection.

## 2. Materials and Methods

### 2.1. Study Design and Patient Selection

This retrospective, single-center observational study was conducted in the Emergency Department of the Municipal Clinical Hospital, Timișoara, Romania. It included all consecutive adult patients admitted with suspected bacterial infection between 1 June 2025 and 31 August 2025, after eligibility screening based on predefined inclusion and exclusion criteria. The cohort therefore comprised adults evaluated in the Emergency Department and subsequently admitted to hospital: all 213 patients included were admitted either to a general ward or directly to the intensive care unit, and none were discharged directly from the Emergency Department.

Patients clinically managed for suspected bacterial infection in the emergency department were eligible if they were adults presenting with a clinical syndrome characterized by signs and symptoms compatible with an acute bacterial infection (e.g., fever, chills, altered general condition, or a clinically identifiable infectious focus), together with laboratory evidence of systemic inflammation (elevated inflammatory biomarkers), and if the treating physician initiated empirical antibiotic therapy before microbiological results were available or made a diagnosis consistent with bacterial infection. The overall clinical assessment incorporated the available clinical, laboratory, imaging, and microbiological findings, together with the treating physician’s judgment.

Patients were excluded when the subsequent Emergency Department evaluation identified an alternative non-infectious diagnosis that better explained the clinical presentation and bacterial infection was no longer retained as the primary working diagnosis. Alternative non-infectious diagnoses included acute cardiovascular, neurological, thromboembolic, metabolic, inflammatory, or other non-infectious conditions, based on the final ED assessment and the available clinical, laboratory, and imaging findings. Absence of microbiological confirmation alone was not considered an exclusion criterion [1,3,4,16].

**Figure 1:**
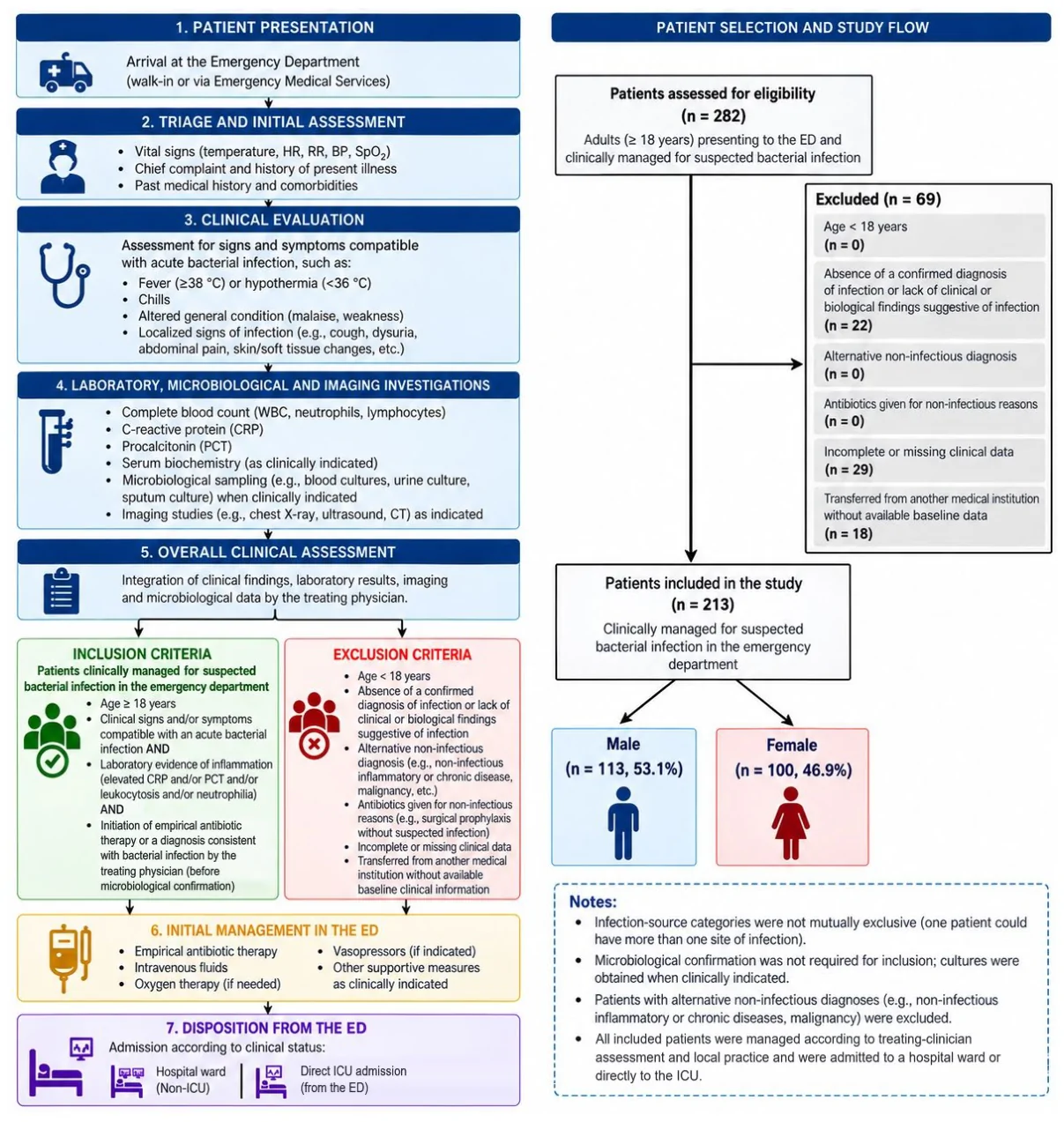
Diagnostic algorithm, patient eligibility assessment, initial management, and study flow of adult patients clinically managed for suspected bacterial infection in the emergency department. The figure illustrates the clinical assessment, inclusion and exclusion criteria, patient selection process, initial therapeutic management, and final hospital disposition.

The diagnostic algorithm and patient selection pathway are illustrated in Figure 1. All consecutive adult patients presenting to the Emergency Department during the study period who met the predefined inclusion criteria were screened for eligibility. Upon presentation to the Emergency Department, all patients underwent triage, clinical evaluation, laboratory investigations including inflammatory biomarkers, and microbiological sampling when clinically indicated. Based on the treating physician’s overall assessment, integrating clinical signs and symptoms compatible with acute bacterial infection, laboratory evidence of inflammation, and the initiation of empirical antibiotic therapy before microbiological confirmation or a diagnosis consistent with bacterial infection, patients were screened for eligibility. Inclusion criteria were: (1) patients aged 18 years or above; (2) clinical signs and/or symptoms compatible with an acute bacterial infection, accompanied by laboratory evidence of inflammation (e.g., elevated C-reactive protein or procalcitonin, leukocytosis, or neutrophilia); (3) complete evaluation in the Emergency Department with all relevant clinical and laboratory data available; and (4) initiation of empirical antibiotic therapy or a diagnosis consistent with bacterial infection made by the treating physician. Patients not meeting the predefined inclusion criteria, those with incomplete clinical records, or those transferred from another institution without baseline clinical information were excluded. Eligible patients subsequently received initial management according to the treating clinician’s assessment and local practice, including empirical antibiotic therapy, intravenous fluid resuscitation, and additional supportive measures when indicated, before admission to a hospital ward or direct admission to the intensive care unit (ICU), according to clinical status. Clinical severity was assessed using vital signs, the Glasgow Coma Scale (GCS), the quick Sequential Organ Failure Assessment (qSOFA) score, inflammatory biomarkers, and the requirement for vasopressor therapy. Vasopressor use was taken as a sign of hemodynamic instability and increased severity of disease, in line with the Sepsis-3 criteria and current recommendations for the management of sepsis [3,4].

### 2.2. Clinical Assessment and Laboratory Measurements

Demographic features, comorbidities, clinical findings, and laboratory parameters, along with treatment interventions, infection source, and patient outcomes, were extracted from electronic medical records.

Venous blood samples were collected from all patients at admission to the Emergency Department under standardized conditions. Hemoglobin concentration, total leukocyte count, neutrophil count, and lymphocyte count were determined in samples of whole blood collected in 2 mL K3EDTA tubes using an automated hematology analyzer (Celltac G MEK-9100; Nihon Kohden, Tokyo, Japan).

For inflammatory biomarker assessment, blood samples were collected in serum separator tubes for C-reactive protein (CRP) and procalcitonin measurements and then centrifuged at 3000 rpm for 10 min. Serum concentrations were analyzed using the mini VIDAS automated immunoassay system. All these steps were carried out in accordance with the guidelines specified by the system’s manufacturer, bioMérieux, Marcy-l’Étoile, France.

The documented source of infection was assigned retrospectively according to the final Emergency Department assessment, based on the available clinical findings, laboratory and microbiological investigations, imaging results, and the treating physician’s overall assessment. Source ascertainment was performed independently by two evaluators. In case of disagreement, the final classification was established by discussion and consensus. Sources were categorized as respiratory, urinary, digestive, cutaneous/soft-tissue, or unknown. Microbiological confirmation was not required for source assignment and was not systematically available; therefore, sources were considered documented or presumed rather than microbiologically confirmed. The proportion of patients with microbiological confirmation could not be reliably determined, because microbiological testing was performed according to clinical indication rather than by study protocol and was not systematically available for the entire cohort. Infection-source categories were not mutually exclusive, and patients could have more than one documented source.

Treatment interventions were recorded during the Emergency Department stay, from the initial ED assessment until disposition to the hospital ward or directly to the intensive care unit. Vasopressor therapy was defined as the administration of intravenous vasopressor medication for clinically significant hemodynamic instability. Broad-spectrum antibiotic therapy was defined as empirical systemic antibacterial treatment providing coverage against a broad range of clinically suspected bacterial pathogens, selected by the treating physician according to the suspected infection source and clinical severity. Only treatments administered during the ED stay were included; treatments initiated after hospital admission were not considered. Patient outcomes included hospital ward admission, direct ICU admission from the Emergency Department, and in-hospital mortality.

The primary outcome, direct ICU admission from the Emergency Department (hereafter, direct ICU admission), was defined as admission to the intensive care unit directly from the Emergency Department following the initial ED assessment and management. Patients initially admitted to a general ward and subsequently transferred to the ICU during hospitalization were not considered to have met this outcome. The decision for direct ICU admission was made according to the treating clinicians’ assessment of clinical severity and need for intensive monitoring or organ-supportive therapy, within the local institutional admission practices and available ICU capacity.

The qSOFA score was calculated retrospectively using the first available values recorded during the initial Emergency Department assessment, rather than the worst values recorded during the subsequent ED stay. The score was based on respiratory rate ≥ 22/min, systolic blood pressure ≤ 100 mmHg, and Glasgow Coma Scale score < 15, with one point assigned for each criterion.

### 2.3. Statistical Analysis

*Software and data handling*

All statistical analyses were performed using R version 4.6.0 (24 April 2026; R Foundation for Statistical Computing, Vienna, Austria). The versions of all packages used in the analysis are reported in Supplementary Table S1. The analysis dataset included all 213 eligible patients, with complete data for every study variable; therefore, no imputation was required. Prior to analysis, the dataset was verified against a predefined data dictionary specifying the permitted codes, units and plausible value ranges of each variable, and was subjected to internal consistency checks covering the leukocyte differential, blood pressure values and the components of the severity score. The neutrophil-to-lymphocyte ratio was verified against the corresponding absolute neutrophil and lymphocyte counts in every patient. In one patient, the absolute neutrophil count was derived from the measured neutrophil percentage and the total leukocyte count. Figures were produced using the ggplot2 and patchwork packages, with a colourblind-safe Okabe–Ito palette.

*Exposure and reference categories*

Biological sex was the exposure variable throughout the analysis. Male sex was set as the reference category, so that all odds ratios are expressed as female versus male.

*Descriptive statistics*

The distribution of continuous variables was examined using the Shapiro–Wilk test. As all continuous variables departed significantly from normality, they are reported as median (interquartile range, IQR), which is the primary presentation throughout. The corresponding means ± standard deviations, reported to allow comparison with previously published cohorts, are given in Supplementary Tables S2 and S3. Categorical variables are reported as absolute frequencies and percentages.

*Unadjusted comparisons*

Continuous variables were compared between female and male patients using the Mann–Whitney U test, with the rank-biserial correlation reported as a measure of effect size, positive values indicating higher values in female patients. Its 95% confidence interval was obtained by percentile bootstrap with 2000 resamples stratified by sex, using a fixed random seed (20260821). Categorical variables were compared using Pearson’s chi-squared test without continuity correction, or Fisher’s exact test when any expected cell frequency was below five; in the event, no comparison required the exact test, the smallest expected cell frequency across the analysis being 7.5. Unadjusted odds ratios with 95% confidence intervals are reported alongside *p*-values throughout. They were obtained from logistic regression models containing sex as the only covariate, which for a binary exposure is algebraically identical to the estimate from the corresponding 2 × 2 table; 95% confidence intervals were derived from the Wald standard error of the log odds ratio, as were those for the adjusted models. Sources of infection were recorded as non-mutually exclusive categories and were consequently analysed as separate binary variables; percentages across infection sources therefore sum to more than 100%. Because the categories overlap, the source-specific models are separate binary contrasts rather than a partition of the cohort; the resulting odds ratios are not comparable as competing proportions.

*Analytical framework*

Because sex was treated as the exposure of interest rather than as one predictor among many, covariates were selected a priori rather than on the strength of their association with sex in the present sample. All models are adjusted associational models: they describe the association between sex and the variable under study that remains after conditioning on the covariates, and they are not intended to identify a causal, still less a specifically biological, effect of sex. Age and comorbidity burden were entered as adjustment variables. Age cannot be a consequence of sex, although women and men differ in age in this cohort as a consequence of differential survival to older age, and age is a strong determinant of both the clinical characteristics and the outcomes examined here. Comorbidity burden describes the case-mix with which patients presented, so that the adjusted estimates contrast women and men of comparable age and chronic disease burden; it was retained in the primary model although its distribution did not differ significantly between the sexes. Comorbidity burden may itself partly reflect sex-related differences in health status and in access to care accumulated over the life course, and conditioning on it may therefore remove part of an indirect association between sex and outcome. The unadjusted estimate is accordingly best read as the total association and the adjusted estimate as the association remaining after adjustment; both are reported throughout. The assumed relations among sex, age, comorbidity burden and the outcomes are set out as a directed acyclic graph in Supplementary Figure S1. Illness severity at presentation, source of infection and the therapeutic interventions delivered in the Emergency Department lie between sex and outcome in that graph; conditioning on them would change the quantity being estimated, and they were therefore reported descriptively rather than entered as covariates. Comorbidity burden was summarised as the total number of documented chronic conditions recorded in the electronic medical record, one point being counted for each of the seven categories listed in Table 1 (cardiovascular, pulmonary, renal, cerebral, liver and malignant disease, and diabetes mellitus), each condition counted once irrespective of its severity or of the number of individual diagnoses recorded within that category; the possible range was therefore 0 to 7 and the observed range 0 to 6. An unweighted count was preferred to a weighted index for three reasons. First, weighted indices such as the Charlson comorbidity index require graded diagnostic detail that could not be retrieved consistently from the retrospective record. Second, the count consumes a single degree of freedom, whereas entering the seven conditions separately would have required seven; with 35 events in the primary outcome model the unweighted count preserves approximately twelve events per estimated parameter, against approximately four if the conditions were entered individually. Third, the adequacy of the unweighted linear specification was examined empirically rather than assumed, as described below.

The analysis plan—the designation of the primary outcome, the covariate set for each model and the three-level analytical hierarchy described below—was defined and documented after data collection but before any model was fitted. Throughout this article, the term “planned” therefore denotes specification before model fitting and does not imply a prospectively registered protocol.

*Adjusted analyses*

Two sets of adjusted analyses were planned before model fitting, corresponding to the two components of the study question.

First, in order to establish whether the sex-related differences observed in clinical characteristics were independent of the age difference between groups, logistic regression models of the form characteristic ~ sex + age were fitted for a planned set of four variables: cardiovascular disease, renal disease, and urinary and respiratory sources of infection.

Second, direct ICU admission was designated the primary outcome and analysed by multivariable logistic regression including sex, age and comorbidity count as covariates. In-hospital mortality was analysed as a planned secondary outcome using a more parsimonious model containing sex and age only, in view of the limited number of events (16 deaths, corresponding to eight events per estimated parameter). Vasopressor therapy was treated as a therapeutic intervention rather than as a study outcome and is reported descriptively. Both outcome models were assessed for separation and quasi-separation and, irrespective of the result, were additionally re-estimated by Firth’s penalized likelihood as a sensitivity analysis, in view of the limited number of events, using the logistf package. The primary model was additionally re-estimated without the comorbidity count, so that the estimate for sex adjusted for age alone could be compared with the fully adjusted estimate (Supplementary Table S4).

Before interpretation, we examined the primary model for collinearity using variance inflation factors, and we tested the adequacy of a linear age effect in both outcome models with a likelihood ratio test against a model including an additional quadratic age term. The assumption that comorbidity count acts linearly was examined in the primary model by the same likelihood ratio approach and by refitting the model with the count entered as four categories (0–1, 2, 3 and 4 or more conditions).

After the planned analyses had been completed, a sex-by-age interaction term was added to each outcome model as a post hoc check for effect modification. Because the number of events supports such a test poorly, the stability of the result was assessed by estimating the age effect separately within each sex and by repeating the test with each death omitted in turn. These post hoc analyses are exploratory and hypothesis-generating, carry no inferential weight, and are reported in full in Supplementary Text S1 and Supplementary Table S5.

*Multiplicity and interpretation*

The analyses were organised in three levels. The primary analysis was restricted to the model for direct ICU admission. The model for in-hospital mortality and the four age-adjusted analyses of clinical characteristics were planned secondary analyses. All unadjusted comparisons were exploratory. Analyses conducted after the analysis plan had been executed, namely the tests for effect modification by age, are identified as post hoc wherever they appear and carry no inferential weight.

The primary analysis consisted of a single test, that of the association between sex and direct ICU admission, so no adjustment for multiplicity was required within it. The four age-adjusted comparisons of clinical characteristics form a single family of related hypotheses and are interpreted inferentially; the Benjamini–Hochberg procedure was therefore applied within that family, and the adjusted values are reported alongside the unadjusted ones. The model for in-hospital mortality addresses a different question and constitutes a family of one, so no adjustment applies to it. The exploratory comparisons are reported without adjustment and are interpreted descriptively rather than inferentially, so that no correction is implied by their *p*-values; for transparency, the effect of applying the same procedure across all of them is nevertheless reported in the Results.

*Precision*

No a priori sample size calculation was performed, since the study included all eligible patients presenting during the study period. Study precision is therefore characterised by the confidence intervals obtained for the effect of sex, which are interpreted as the range of effects compatible with the data. For the two study outcomes, where neither association reached statistical significance, the range of effects that the data cannot exclude is stated explicitly in the Results. Throughout, a non-significant finding is reported as an absence of precise evidence for a sex-related difference rather than as evidence of equivalence between groups.

All tests were two-sided and a *p*-value below 0.05 was considered statistically significant.

## 3. Results

### 3.1. Baseline Demographic and Clinical Characteristics

**Figure 2:**
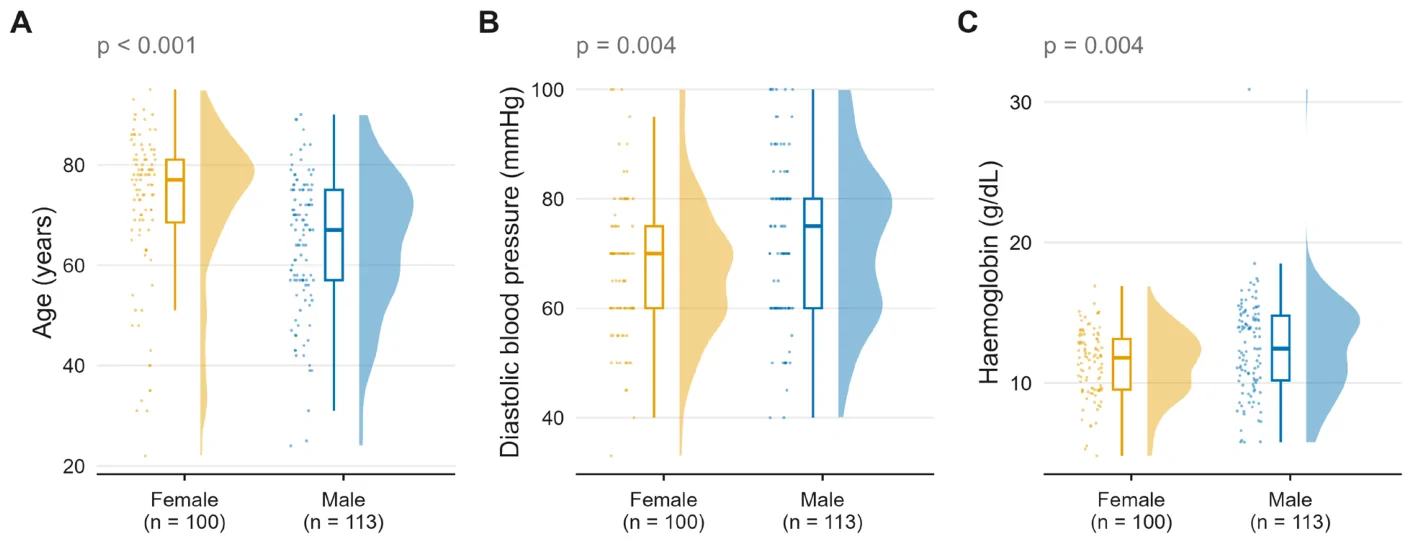
Distribution of age, diastolic blood pressure and haemoglobin according to sex among patients clinically managed for suspected bacterial infection in the emergency department. (**A**) Age. (**B**) Diastolic blood pressure. (**C**) Haemoglobin. Each panel shows the kernel density of the distribution, a box plot marking the median and interquartile range with whiskers extending to 1.5 times the interquartile range, and the individual observations. Groups were compared using the Mann–Whitney U test.

Of 282 adults assessed for eligibility during the study period, 69 were excluded: 22 because the subsequent ED evaluation did not support suspected bacterial infection, owing to a lack of clinical or biological findings suggestive of infection (no patient was excluded because of an alternative non-infectious diagnosis); 29 because of incomplete or missing clinical data; and 18 because they had been transferred from another institution without available baseline data. A total of 213 patients with clinically suspected bacterial infection presenting to the Emergency Department were therefore included in the analysis; 100 were female (46.9%) and 113 were male (53.1%). The selection process is shown in Figure 1. Complete data were available for all study variables in every patient. Female patients were older than male patients, with a median age of 77.0 (68.5–81.0) years compared with 67.0 (57.0–75.0) years (*p* < 0.001; rank-biserial correlation 0.392, 95% CI 0.245–0.542). Conversely, male patients presented with higher diastolic blood pressure, 75 (60–80) mmHg versus 70 (60–75) mmHg (*p* = 0.004). No differences were observed between the sexes in systolic blood pressure (*p* = 0.823), oxygen saturation on room air (*p* = 0.339), body temperature (*p* = 0.455), Glasgow Coma Scale score (*p* = 0.992) or qSOFA score (*p* = 0.885). Baseline characteristics are summarised in Table 2, and the distributions of age and diastolic blood pressure are shown in Figure 2A,B.

### 3.2. Comorbidity Profile

**Figure 3:**
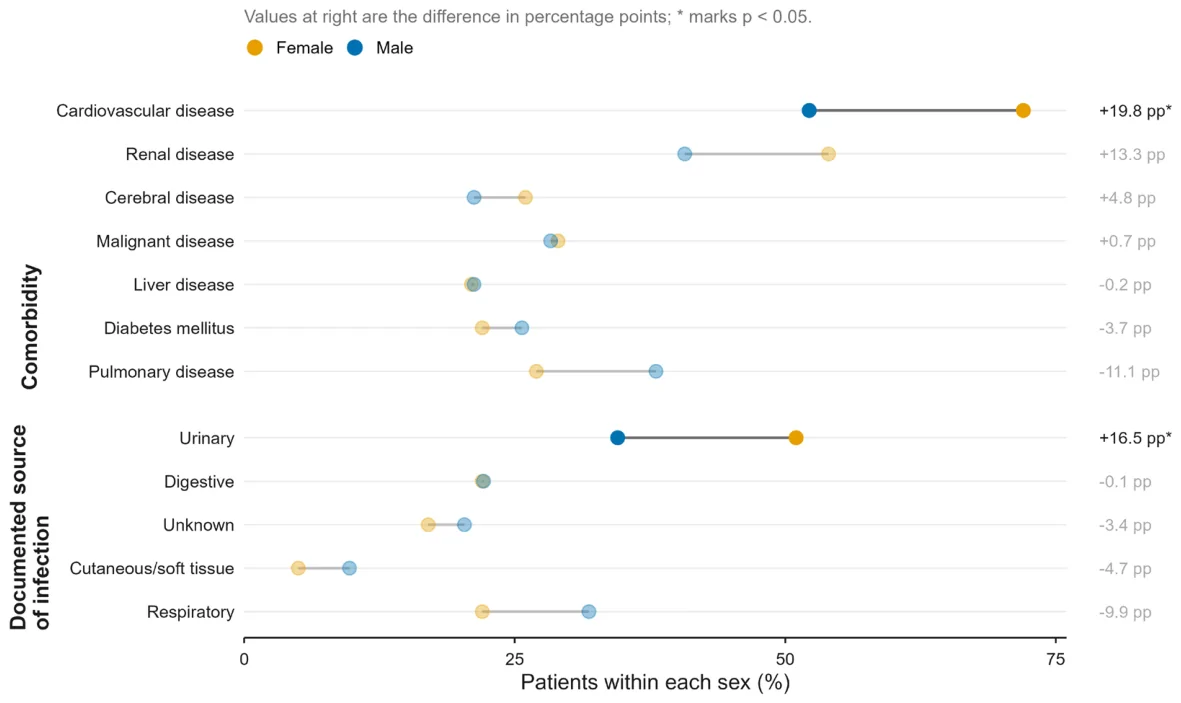
Prevalence of comorbidities and of documented sources of infection according to sex. Each pair of points represents the percentage of female and of male patients with the characteristic, joined by a line whose length is the absolute difference between them; this difference is given at the right in percentage points. Percentages are calculated within each sex. Rows are ordered from the largest excess among women to the largest excess among men, and characteristics for which the sexes differed at *p* < 0.05 on Pearson’s chi-squared test are shown at full intensity. Sources of infection are not mutually exclusive.

Cardiovascular disease was the most frequent comorbidity in the cohort and was more common among female than male patients (72.0% versus 52.2%; odds ratio 2.35, 95% CI 1.33–4.17; *p* = 0.003). Renal disease was also more frequent in women (54.0% versus 40.7%), although the difference did not reach statistical significance (odds ratio 1.71, 95% CI 0.99–2.94; *p* = 0.052). Pulmonary disease was more frequent among men (38.1% versus 27.0%; *p* = 0.087). The prevalence of diabetes mellitus, cerebral disease, liver disease and malignant disease was comparable between the sexes (all *p* ≥ 0.413). The total number of documented comorbidities did not differ significantly between groups, with a median of 2 (2–3) in female and 2 (1–3) in male patients (*p* = 0.162); across the cohort the count ranged from 0 to 6. Comorbidities are presented in Table 1 and, together with the sources of infection, in Figure 3.

### 3.3. Emergency Department Treatment Interventions

No statistically significant difference in the treatment delivered in the Emergency Department was detected between women and men. Supplemental oxygen was administered to 28.0% of female and 28.3% of male patients (*p* = 0.959). Vasopressor therapy was given to 15.0% of female and 23.0% of male patients (odds ratio 0.59, 95% CI 0.29–1.19; *p* = 0.139). Broad-spectrum antibiotics were administered to 88.0% of female and 87.6% of male patients (*p* = 0.931). Intravenous fluids were administered to every patient in the cohort and were therefore not amenable to between-group comparison. Table 3 shows treatment interventions.

### 3.4. Laboratory Parameters at Admission

Haemoglobin concentration was lower in female than in male patients, with a median of 11.79 versus 12.45 g/dL (*p* = 0.004; rank-biserial correlation −0.227, 95% CI −0.373 to −0.076), as shown in Figure 2C. Median C-reactive protein concentration was 132.9 mg/L in women and 184.4 mg/L in men, with no significant difference between them (*p* = 0.203). Total leukocyte, neutrophil and lymphocyte counts, the neutrophil-to-lymphocyte ratio and procalcitonin concentration were likewise comparable between the sexes (all *p* ≥ 0.369). Laboratory parameters are presented in Table 4.

### 3.5. Infection Source Distribution

A documented urinary source of infection was the most frequent in the cohort and was more common among female than male patients (51.0% versus 34.5%; odds ratio 1.97, 95% CI 1.14–3.43; *p* = 0.015). A documented respiratory source was more frequent among men (31.9% versus 22.0%), without reaching statistical significance (*p* = 0.107). Digestive, cutaneous or soft-tissue and unknown sources were distributed similarly between the sexes (all *p* ≥ 0.191). Sources of infection were recorded as non-mutually exclusive categories, and percentages therefore sum to more than 100%. More than one source was documented in 37 patients (17.4%): two sources in 36 patients and three sources in one. The distribution of infection sources is presented in Table 5 and in Figure 3.

### 3.6. Clinical Outcomes

Direct ICU admission occurred in 14 of 100 female patients (14.0%) and in 21 of 113 male patients (18.6%; odds ratio 0.71, 95% CI 0.34–1.49; *p* = 0.368). In-hospital mortality occurred in 7 of 100 women (7.0%) and in 9 of 113 men (8.0%; odds ratio 0.87, 95% CI 0.31–2.43; *p* = 0.790). Neither comparison provided precise evidence of a sex-related difference; the confidence intervals were wide, and clinically relevant differences cannot be excluded on these data. Ward admission is the exact complement of direct ICU admission in this cohort and is therefore not reported separately. Clinical outcomes are presented in Table 6.

### 3.7. Clinical Characteristics After Adjustment for Age

**Figure 4:**
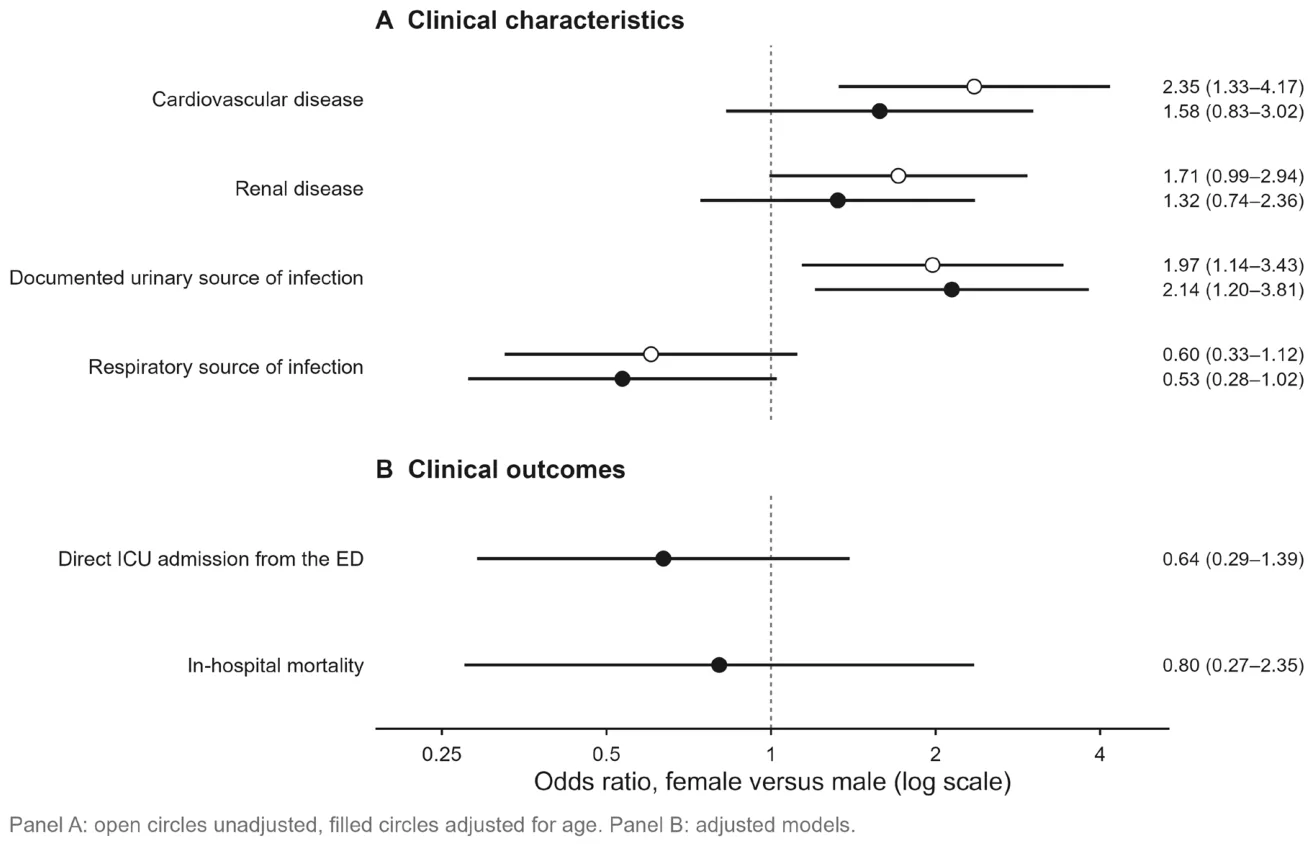
Odds ratios for female versus male sex, obtained from logistic regression models. (**A**) Clinical characteristics, shown before (open circles) and after (filled circles) adjustment for age. (**B**) Clinical outcomes, adjusted for age and number of comorbidities (direct ICU admission from the Emergency Department) or for age alone (in-hospital mortality). Horizontal lines denote 95% confidence intervals and the dashed vertical line marks an odds ratio of 1. The horizontal axis is on a logarithmic scale. ED, Emergency Department; ICU, intensive care unit.

Because female patients were older than male patients, the sex-related differences observed in clinical characteristics were re-examined after adjustment for age. The excess of cardiovascular disease among women was attenuated and no longer significant after adjustment, the odds ratio falling from 2.35 (95% CI 1.33–4.17) to 1.58 (95% CI 0.83–3.02), *p* = 0.166, while age itself was associated with cardiovascular disease (odds ratio per year 1.07, 95% CI 1.05–1.10; *p* < 0.001). A similar pattern was observed for renal disease, where the odds ratio fell from 1.71 (95% CI 0.99–2.94) to 1.32 (95% CI 0.74–2.36), *p* = 0.341, with age again independently associated (odds ratio per year 1.04, 95% CI 1.01–1.06; *p* = 0.001). In contrast, the predominance of a documented urinary source of infection among women was not attenuated but slightly strengthened after adjustment, from 1.97 (95% CI 1.14–3.43) to 2.14 (95% CI 1.20–3.81), *p* = 0.010, and age was not associated with a documented urinary source (odds ratio per year 0.99, 95% CI 0.97–1.01; *p* = 0.315). The lower frequency of a respiratory source among women did not reach statistical significance after adjustment (odds ratio 0.53, 95% CI 0.28–1.02; *p* = 0.059). Because the urinary and respiratory categories are not mutually exclusive, these two models were fitted separately and estimate distinct, partly overlapping contrasts rather than components of a single partition of the cohort. After Benjamini–Hochberg adjustment across these four secondary comparisons, the association with a documented urinary source of infection remained significant (adjusted *p* = 0.039), while the adjusted values for the remaining three characteristics ranged from 0.118 to 0.341. These results are presented in Table 7 and Figure 4A.

### 3.8. Adjusted Analysis of Clinical Outcomes

In the multivariable model for the primary outcome, female sex was not associated with direct ICU admission after adjustment for age and number of comorbidities (odds ratio 0.64, 95% CI 0.29–1.39; *p* = 0.257). Age was not associated with the outcome (odds ratio per year 1.00, 95% CI 0.97–1.04; *p* = 0.765), whereas a higher number of comorbidities was (odds ratio per additional condition 1.37, 95% CI 1.02–1.86; *p* = 0.038); the latter is a covariate rather than the exposure under study and is reported for completeness. In the planned secondary model for in-hospital mortality, female sex was likewise not associated with the outcome after adjustment for age (odds ratio 0.80, 95% CI 0.27–2.35; *p* = 0.690). Neither model showed evidence of separation; both were estimated by standard maximum likelihood, as specified. These are adjusted associational estimates: the unadjusted odds ratios reported in Section 3.6 describe the total association between sex and each outcome, whereas the estimates presented here describe the association remaining after conditioning on age and, for direct ICU admission, on comorbidity burden. Results are presented in Table 8 and Figure 4B.

In the primary model, variance inflation factors ranged from 1.08 to 1.25, well below the conventional threshold of 5 for problematic collinearity between sex, age and comorbidity count. The assumption of a linear effect of age was not rejected for either outcome (*p* = 0.942 for direct ICU admission and *p* = 0.576 for in-hospital mortality).

The assumption that comorbidity count acts linearly on the odds of the primary outcome was examined in a further sensitivity analysis. Neither the addition of a quadratic term (*p* = 0.270) nor a test for departure from linearity with the count entered as four categories (0–1, 2, 3 and 4 or more; *p* = 0.178) indicated a violation, and the linear specification was retained. The primary model was also re-estimated with the comorbidity count omitted. The estimate for female sex was essentially unchanged, at 0.63 (95% CI 0.29–1.37) against 0.64 (95% CI 0.29–1.39) in the primary model, the two log odds ratios differing by less than 1%. The conclusion regarding sex therefore does not depend on the decision to adjust for comorbidity burden (Supplementary Table S4).

As a sensitivity analysis, both models were re-estimated by Firth’s penalized likelihood. No odds ratio differed from the corresponding maximum likelihood estimate by more than 4%; for female sex the penalized estimates were 0.65 (95% CI 0.30–1.40) for direct ICU admission and 0.84 (95% CI 0.28–2.36) for in-hospital mortality. Firth-penalized estimates were thus similar to the conventional maximum-likelihood estimates, suggesting that complete or quasi-complete separation was unlikely to have materially distorted the point estimates. However, the limited number of outcome events continued to constrain the precision of all effect estimates.

The precision of these estimates was limited by the number of events. For direct ICU admission, based on 35 events, the confidence interval for female sex was compatible with anything from a 71% reduction to a 39% increase in the odds of the outcome. For in-hospital mortality, based on 16 events, the corresponding range extended from a 73% reduction to a 135% increase. The absence of a statistically significant association should therefore be interpreted as an absence of precise evidence for a sex-related difference, and not as evidence that outcomes are equivalent between the sexes.

In a post hoc, exploratory analysis, the sex-by-age interaction term was not significant for direct ICU admission (*p* = 0.323) and reached nominal significance for in-hospital mortality (*p* = 0.024). This analysis was not part of the analysis plan and is hypothesis-generating: it rests on 16 events and three parameters, and it was not robust to the leave-one-out omission of individual deaths. It carries no inferential weight and does not provide evidence of effect modification. The full results, including the sex-specific age effects and the leave-one-out analysis, are reported in Supplementary Text S1 and Supplementary Table S5.

The unadjusted comparisons reported in Section 3.1, Section 3.2, Section 3.3, Section 3.4, Section 3.5 and Section 3.6 are descriptive and were not corrected for multiplicity. For transparency, applying the Benjamini–Hochberg procedure across all 32 of them leaves four of the differences significant at the 5% level: age, diastolic blood pressure, cardiovascular disease and haemoglobin. The unadjusted difference in documented urinary source of infection does not survive correction across this larger exploratory family; the corresponding age-adjusted comparison, which belongs to the planned secondary family and carries the inferential weight, does (Section 3.7).

## 4. Discussion

The present study investigated sex-related differences in demographic characteristics, comorbidities, infection source, laboratory findings, treatment requirements, and short-term outcomes among 213 adult patients clinically managed for suspected bacterial infection in the emergency department. Female patients were older than male patients (median 77.0 versus 67.0 years; *p* < 0.001) and had a higher unadjusted prevalence of cardiovascular disease (72.0% versus 52.2%; *p* = 0.003), whereas male patients presented with higher diastolic blood pressure (75 versus 70 mmHg; *p* = 0.004). A documented urinary source of infection was the most frequent in the cohort and was more common among women (51.0% versus 34.5%; odds ratio 1.97, 95% CI 1.14–3.43), whereas a documented respiratory source was more frequent among men without reaching statistical significance (31.9% versus 22.0%; *p* = 0.107). No statistically significant difference between women and men was detected in the treatment delivered in the Emergency Department, in direct ICU admission (14 of 100 versus 21 of 113; odds ratio 0.71, 95% CI 0.34–1.49) or in in-hospital mortality (7 versus 9; odds ratio 0.87, 95% CI 0.31–2.43); the confidence intervals were wide, however, and clinically relevant differences cannot be excluded.

One of the most notable findings was the significantly higher age of female patients. Similar results have been described in other investigations of sex-related differences in sepsis and infection. Wanrooij et al. [13] found that females who presented to the emergency department with sepsis were generally older than males, while Mewes et al. noted a higher prevalence of advanced age among female patients with sepsis and septic shock [9]. The finding may be a reflection of increasing life expectancy for women and the growing burden of age-related chronic diseases. Furthermore, aging is related to immunosenescence, which is the aging of the immune system, leading to impaired immune function, chronic low-grade inflammation, and increased susceptibility to infections. These factors may influence both the presentation of disease and the requirements of treatment [8,9].

Compared with prospective or more highly standardized observational studies of patients with suspected infection, in which treatment exposure has been defined using prespecified timing, dose, or observation windows, treatment variables in the present study were retrospectively extracted from routine Emergency Department documentation. For example, previous studies have evaluated time to antibiotic administration from ED triage and have recorded the type and timing of vasopressor administration over predefined periods [17,18]. Other studies have similarly used predefined time intervals from ED presentation or recognition of sepsis to antibiotic administration and vasopressor initiation [19]. In the present study, supplemental oxygen, vasopressor therapy, and broad-spectrum antibiotic treatment were therefore interpreted as descriptive indicators of initial ED management rather than as standardized measures of treatment intensity. Variability in clinical decision-making, treatment timing, and documentation may have influenced the observed frequencies and limits direct comparison with studies using prospectively defined treatment protocols.

In the present cohort, qSOFA scores at Emergency Department presentation did not differ between women and men, with a median score of 0 (IQR 0–1) in both groups (*p* = 0.885). This finding suggests that, despite the significantly older age and higher prevalence of cardiovascular disease observed among women, there was no detectable sex-related difference in acute physiological derangement as captured by the qSOFA score. This observation is relevant because previous studies have demonstrated that qSOFA is associated with adverse outcomes among patients presenting to the Emergency Department with suspected infection. In a large cohort of 11,205 ED patients with suspected infection, qSOFA-positive patients had substantially higher rates of in-hospital mortality and prolonged ICU stay than qSOFA-negative patients, although the positive predictive value for mortality was limited [20]. Similarly, Singer et al. reported increasing rates of mortality and ICU admission with increasing qSOFA scores among more than 22,000 ED patients [21]. These studies support the value of qSOFA as a marker of acute illness severity, but they did not specifically establish a sex-related difference in qSOFA among patients with suspected bacterial infection. Our finding therefore adds a complementary perspective, suggesting that the observed differences between women and men in age, comorbidity profile, and infection source were not accompanied by a difference in baseline qSOFA-defined severity. Importantly, qSOFA was retained in the present study as a descriptive measure of initial clinical status and was not used as an independent predictor in the final outcome models; therefore, no conclusion regarding its independent association with direct ICU admission or mortality can be drawn from our data. Interestingly, studies using the full SOFA score have reported sex-related differences in specific domains of organ dysfunction, particularly renal, hepatic, and coagulation parameters, despite the absence of consistent differences in short-term mortality [22]. In contrast, the absence of a sex difference in qSOFA in our cohort suggests that such differences may not necessarily be reflected in this simplified bedside severity score.

Cardiovascular disease was the most common comorbidity in our cohort and was significantly more common among female patients (72.0% vs. 52.2%; *p* = 0.003). This is probably related to the fact that the women in our study were older; prevalence of cardiovascular disease increases with age. Similar findings were reported previously when it was observed that women presenting to the Emergency Department with sepsis were generally older and exhibited a distinct comorbidity profile compared with men, including a higher burden of chronic cardiovascular conditions [13]. Likewise, Hajji et al. demonstrated that older female patients with sepsis frequently presented with a greater prevalence of cardiovascular comorbidities, which influenced clinical presentation and management strategies [15].

In earlier sepsis cohorts, however, rates of cardiovascular disease between the sexes were either similar or showed a predominance of male patients, further underlining differences in study populations, age distributions, and inclusion criteria [8,9]. Cardiovascular comorbidities may be clinically relevant in this context, as pre-existing cardiovascular disease can reduce physiological reserve and may influence the ability to tolerate the hemodynamic stress associated with systemic infection [23]. However, the present study cannot establish a causal relationship between cardiovascular comorbidity and subsequent clinical deterioration. Moreover, the observed higher prevalence of cardiovascular disease among women should be interpreted in the context of their significantly greater age, particularly because the age-adjusted analysis attenuated this association.

The distribution of the sources of infections revealed important sex-related differences in our cohort. A documented urinary source of infection was the most frequent in the cohort and was significantly more prevalent in female than in male patients (51.0% vs. 34.5%, *p* = 0.015). This difference refers to the documented source of infection, assigned retrospectively from the final Emergency Department assessment; because microbiological confirmation was neither required for source assignment nor systematically available, it should not be interpreted as a microbiologically verified sex difference in urinary tract infection. With this qualification, the finding is in line with the known epidemiology of urinary tract infections and previous studies that have reported a marked female predominance in urinary tract infections. Foxman demonstrated that urinary tract infections represent one of the most common bacterial infections among women, with more than half of all women experiencing at least one episode during their lifetime due to anatomical and physiological factors favoring bacterial colonization and ascending infection [24]. Similarly, Dias et al. noted that women are disproportionately affected by urinary tract infections due to sex-specific anatomical, hormonal, and microbiological factors that influence the susceptibility to bacterial infection [10]. Furthermore, Wanrooij et al. noted that female patients presenting to the Emergency Department with sepsis mostly had urinary tract infections as the primary source of infection, which supports the existence of sex-related differences in the distribution of infection sources [13]. In our cohort, the predominance of a documented urinary source of infection among women was not attenuated but slightly strengthened after adjustment, from 1.97 (95% CI 1.14–3.43) to 2.14 (95% CI 1.20–3.81), *p* = 0.010, and age was not associated with a documented urinary source (odds ratio per year 0.99, 95% CI 0.97–1.01; *p* = 0.315).

A documented respiratory source of infection was more frequent among male than among female patients in our study (31.9% vs. 22.0%), although the difference was not statistically significant (*p* = 0.107). This finding is consistent with other studies in which men were reported to bear a higher burden of respiratory infections. For example, Falagas et al. showed that, compared with women, men have both higher incidence and severity of respiratory infections; this difference may be related to variations in smoking habits, pulmonary comorbidities, environmental exposures, and host immune responses between the two sexes [11]. Similar observations were reported by Wanrooij et al. and Pepe et al., both of whom found respiratory infections to be more common among male patients presenting with sepsis or suspected infection in Emergency Department settings [12,13]. Experimental evidence further suggests that sex hormones influence innate and adaptive immune responses, contributing to sex-specific susceptibility to respiratory pathogens, which may partly account for the numerically higher, although not statistically significant, frequency of a documented respiratory source among men in our cohort [25,26].

Furthermore, vasopressor use in clinical practice is generally considered a sign of hemodynamic instability and therefore greater severity of illness in patients with infection and sepsis [3,4]. Our results showed that administration of vasopressor therapy did not differ significantly between women and men. Mewes et al. noted sex-related differences in organ dysfunction and therapeutic requirements among patients with sepsis and septic shock, but there was no consistent difference in mortality outcomes between women and men [9]. Wanrooij et al. demonstrated that female patients presenting to the Emergency Department with sepsis often exhibited a different clinical phenotype and comorbidity profile, although mortality rates remained comparable between sexes [13].

Laboratory analyses showed marked inflammatory activation in both sexes. Median C-reactive protein concentration was 132.9 mg/L in women and 184.4 mg/L in men, with no significant difference between them (*p* = 0.203). Total leukocyte, neutrophil and lymphocyte counts, the neutrophil-to-lymphocyte ratio and procalcitonin concentration were likewise comparable between the sexes (all *p* ≥ 0.369). Sex-related differences in inflammatory responses have been described in other studies. Rio et al. [6] and Araújo et al. [7] stated that biological sex has an influence on innate and adaptive immune responses through genetic and hormonal mechanisms, leading to cytokine production, leukocyte activation, and inflammatory signaling pathway differences during infection. In particular, estrogens have been associated with enhanced immune responsiveness and effective pathogen clearance, while testosterone has been predominantly immunosuppressive, possibly contributing to different inflammatory profiles between women and men. Our findings are also supported by Trebuian et al. [14], who showed that inflammatory biomarkers play a critical role in assessing disease severity and prognosis in patients with septic conditions. Likewise, Hajji et al. [15] showed sex- and age-related differences in the inflammatory response of septic and septic shock patients and therefore suggested that biological sex could contribute to variation in biomarker expression and immune activation. We found no statistically significant sex-related differences in the inflammatory biomarkers evaluated in this cohort. Therefore, our results do not provide sufficient evidence for a sex-specific inflammatory profile. Larger prospective studies are required to determine whether clinically relevant differences exist.

The findings of the revised multivariable analysis are broadly consistent with the heterogeneous literature regarding the prognostic role of biological sex in infection and sepsis. In our cohort, female sex was not independently associated with direct ICU admission after adjustment for age and comorbidity burden (OR 0.64, 95% CI 0.29–1.39; *p* = 0.257), and it was likewise not independently associated with in-hospital mortality after adjustment for age (OR 0.80, 95% CI 0.27–2.35; *p* = 0.690). These estimates were essentially unchanged when the comorbidity count was omitted from the model (odds ratio for female sex 0.63, 95% CI 0.29–1.37), so the conclusion regarding sex does not depend on that adjustment decision (Supplementary Table S4). These results are consistent with the large study by Wernly et al., which included 17,146 critically ill patients with sepsis and found identical ICU mortality in women and men (10% vs. 10%), with no significant association between sex and ICU mortality after multivariable adjustment [27]. Similarly, a systematic review and meta-analysis including 80,520 critically ill patients found no clear sex-based difference in adjusted hospital mortality (OR 1.02, 95% CI 0.79–1.32) or ICU mortality (OR 1.19, 95% CI 0.79–1.78), although the certainty of evidence was considered very low [28]. These findings also support the concept that age, comorbidity burden, and acute illness characteristics may collectively have greater prognostic relevance than biological sex alone.

More recent evidence further emphasizes the heterogeneity of sex-related associations with outcomes. A 2026 systematic review and meta-analysis of 18 Western European studies involving 671,861 patients concluded that biological sex was not a robust independent predictor of short-term mortality, despite identifying consistent differences in infection sites, with urinary tract infections predominating among women and pneumonia among men [29]. This pattern closely parallels our findings, in which a documented urinary source of infection was more frequent among women and remained significantly associated with female sex after age adjustment. Importantly, the contemporary literature suggests that sex-related differences in infection presentation may be more readily detectable in infection source and clinical phenotype than in short-term mortality, supporting our observation that differences in presentation do not necessarily translate into differences in direct ICU admission or survival.

A higher comorbidity burden was also associated with direct ICU admission (OR 1.37 per additional condition, 95% CI 1.02–1.86; *p* = 0.038), whereas sex and age were not. This estimate carries three reservations. Comorbidity burden entered the model as an adjustment variable rather than as an exposure of interest, and its coefficient was not the subject of the primary analysis; the confidence interval is compatible with an effect as small as 1.02 per condition; and the association appears step-like rather than graded, the crude rate of direct ICU admission rising from 5.4% among patients with none or one condition to 22.2% among those with two and not increasing further (Supplementary Table S6). With 35 events the data cannot distinguish a linear from a threshold relation, and the per-condition odds ratio should therefore be read as a summary of an overall trend rather than as the effect of each additional condition. The association is nonetheless compatible with the broader prognostic literature, in which comorbidities are commonly incorporated among the major adjustment domains when evaluating the independent prognostic effect of sex. A systematic review for example, specifically identified age, severity of illness, and comorbidities among the important factors that should be considered when assessing sex as a prognostic factor in sepsis [28].

The present findings should nevertheless be interpreted in light of the relatively small number of outcome events. The model for direct ICU admission included 35 events and the mortality model only 16 deaths, resulting in wide confidence intervals. Consequently, the absence of a statistically significant association should be interpreted as an absence of precise evidence for a sex-related difference rather than as evidence of equivalence between women and men. This interpretation is particularly important because the existing literature remains inconsistent: some large observational studies have reported lower mortality among women, particularly in younger patients, whereas other large cohorts and meta-analyses have found no clinically relevant sex-specific mortality difference [28,30].

The exploratory sex-by-age analysis should also be considered in the context of this literature. Although the interaction between sex and age for in-hospital mortality reached nominal significance in our analysis (*p* = 0.024), it was based on only 16 deaths, was not part of the analysis plan, and was not robust to leave-one-out analysis. Therefore, it should not be interpreted as evidence of a true age-dependent sex effect. The full analysis, including the sex-specific age effects and the leave-one-out results, is reported in Supplementary Text S1 and Supplementary Table S5. This cautious interpretation is nevertheless relevant because previous large-scale studies have suggested that age may modify sex-related associations with sepsis outcomes, with differences being more apparent in younger rather than older patients [31,32]. Larger prospective cohorts with adequate numbers of deaths are required to determine whether such interactions are clinically meaningful.

The principal contribution of this study is that sex-related differences observed at presentation did not translate into an independent association with direct ICU admission or in-hospital mortality after adjustment, whereas cumulative comorbidity burden was independently associated with direct ICU admission. This finding adds to the existing literature by emphasizing that sex-related differences in clinical phenotype and infection source should not automatically be interpreted as sex-specific differences in short-term prognosis.

### Study Limitations

The study has several limitations. First, its retrospective single-center design may limit the generalizability of the findings and the ability to determine causal relationships between sex and clinical outcomes.

Second, the relatively small sample size might have limited statistical power to detect subtle sex-related differences, particularly for relatively rare outcomes such as direct ICU admission and in-hospital mortality. Direct ICU admission is a composite measure of clinical severity and healthcare delivery, as the decision to admit a patient directly to the ICU may also be influenced by institutional admission practices, ICU bed availability, clinician judgment, treatment limitations, and transfer practices. In the present study, only direct ICU admissions from the Emergency Department were considered as the primary outcome; subsequent transfers to the ICU after initial ward admission were not included. Therefore, our findings should be interpreted as differences in direct ICU disposition following ED assessment rather than as a comprehensive measure of subsequent critical illness during hospitalization. This limitation is particularly relevant because there were 35 direct ICU admissions and only 16 in-hospital deaths, resulting in relatively wide confidence intervals around the estimates for female sex.

Third, patients were included based on clinical suspicion of bacterial infection, not on microbiologically confirmed infection. This reflects current practice in the Emergency Department and current guidelines that emphasize early recognition and treatment of infection, but it may have led to some degree of diagnostic misclassification [3,4]. Treatment variables were retrospectively extracted from routine Emergency Department documentation and were not assessed using a prospectively standardized treatment protocol. Consequently, differences in treatment selection, timing, and documentation may have influenced the observed frequencies and limit direct comparison with studies using predefined treatment windows. Fourth, microbiological confirmation, distribution of pathogens, and profiles of antimicrobial resistance were not systematically available; thus, a more detailed assessment of potential sex-related differences in infectious etiology was not possible. Likewise, the infection source was assigned from the final Emergency Department assessment and was not microbiologically confirmed. Because asymptomatic bacteriuria is more common in women than in men, urinary findings may have been attributed as the source of infection more readily in women; differential misclassification of this kind could have contributed to the higher frequency of a documented urinary source among women, independently of the age difference between the sexes.

Although qSOFA scores were retrospectively calculated and included in the analyses, comprehensive severity scores such as SOFA and NEWS2 could not be determined because the necessary clinical and laboratory variables were not consistently available in this retrospective cohort. qSOFA should therefore be interpreted primarily as a descriptive indicator of initial clinical severity in the present study. Notably, qSOFA scores did not differ significantly between women and men (median 0 in both groups; *p* = 0.885).

Despite these limitations, the study provides valuable real-world evidence regarding sex-related differences in patients clinically managed for suspected bacterial infection in the emergency department. The inclusion of consecutive patients and the comprehensive assessment of demographic, clinical, laboratory, treatment, and outcome variables strengthen the relevance of the findings and provide a foundation for future prospective investigations. Additionally, the exclusion of patients with incomplete clinical records or unavailable baseline information may have introduced selection bias, potentially limiting the representativeness of the study population.

Furthermore, multivariable logistic regression analyses were performed for the primary outcome of direct ICU admission and the planned secondary outcome of in-hospital mortality. The model for direct ICU admission was adjusted for age and number of comorbidities, whereas the mortality model was adjusted for age because of the limited number of deaths. Female sex was not independently associated with either direct ICU admission or in-hospital mortality, whereas a higher number of comorbidities was independently associated with direct ICU admission. The relatively small number of outcome events nevertheless limited the precision of these estimates and the ability to detect smaller sex-related effects. The adjusted estimates also remain subject to residual confounding. Only age and comorbidity burden were available as adjustment variables, and both were captured coarsely, age as a single linear term and comorbidity burden as an unweighted count of seven conditions. Determinants of both clinical presentation and outcome that were not recorded, among them functional status before admission, frailty, socioeconomic circumstances, the interval between symptom onset and presentation, and the severity of individual chronic conditions, could not be taken into account. Because the models are associational, an unmeasured determinant of this kind could generate or mask an apparent association with sex, and the adjusted estimates should be read with that possibility in mind.

Firth-penalized sensitivity analyses yielded estimates similar to those obtained using conventional maximum likelihood. However, these analyses cannot overcome the fundamental limitation imposed by the small number of outcome events. In addition, the exploratory sex-by-age interaction analysis for in-hospital mortality was not part of the analysis plan and was based on only 16 deaths; although nominally significant, the finding was not robust to leave-one-out analysis and should therefore be considered hypothesis-generating.

Finally, interpret the numerous unadjusted comparisons cautiously because they were primarily descriptive and not initially corrected for multiple testing; although a Benjamini–Hochberg sensitivity analysis was subsequently performed, the possibility of chance findings among exploratory comparisons cannot be completely excluded.

Future multicenter prospective studies are needed to better define the role of biological sex in the clinical course and outcomes of suspected bacterial infections. These studies should include microbiological confirmation, comprehensive severity assessment, and long-term follow-up. Larger sample sizes with sufficient numbers of clinical events would also allow more precise evaluation of sex-related differences in mortality and in ICU admission at any point during hospitalization, as well as more reliable assessment of potential interactions between sex and age. They will also help provide a deeper understanding of sex-specific differences in immune response, hemodynamic adaptation, and treatment requirements, contributing to more individualized approaches to infection management in the Emergency Department.

## 5. Conclusions

This retrospective single-center study identified several sex-related differences among patients clinically managed for suspected bacterial infection in the emergency department. Female patients were older and had a higher unadjusted prevalence of cardiovascular disease, whereas male patients presented with higher diastolic blood pressure. A documented urinary source of infection was more frequent among women and remained significantly associated with female sex after adjustment for age.

No statistically significant differences between women and men were detected in the treatment interventions delivered in the Emergency Department, including supplemental oxygen, vasopressor therapy, and broad-spectrum antibiotics. Similarly, no statistically significant differences were detected in direct ICU admission or in-hospital mortality.

In the adjusted analyses, female sex was not associated with direct ICU admission or in-hospital mortality. However, the relatively small number of outcome events resulted in wide confidence intervals, and these findings should be interpreted as an absence of precise evidence for a sex-related difference rather than as evidence of equivalence between women and men. Firth-penalized sensitivity analyses yielded estimates similar to those obtained using conventional maximum likelihood, but the limited number of outcome events continued to constrain the precision of the effect estimates.

Overall, the findings primarily support descriptive differences in demographic characteristics and documented infection source, particularly the higher frequency of a documented urinary source among women. The study does not provide sufficiently precise evidence to exclude clinically relevant sex-related differences in short-term outcomes. Larger prospective multicenter studies incorporating microbiological confirmation, comprehensive severity assessment, and sufficient numbers of clinical events are warranted to further clarify the independent contribution of biological sex to the management and prognosis of suspected bacterial infection.

## Figures and Tables

**Table 1:** Comorbidities according to sex.

Comorbidity	Female (n = 100)	Male (n = 113)	Effect Size (95% CI)	*p*
Cardiovascular disease, n (%)	72 (72.0)	59 (52.2)	2.35 (1.33–4.17)	0.003
Pulmonary disease, n (%)	27 (27.0)	43 (38.1)	0.60 (0.34–1.08)	0.087
Diabetes mellitus, n (%)	22 (22.0)	29 (25.7)	0.82 (0.43–1.54)	0.532
Renal disease, n (%)	54 (54.0)	46 (40.7)	1.71 (0.99–2.94)	0.052
Cerebral disease, n (%)	26 (26.0)	24 (21.2)	1.30 (0.69–2.46)	0.413
Liver disease, n (%)	21 (21.0)	24 (21.2)	0.99 (0.51–1.91)	0.966
Malignant disease, n (%)	29 (29.0)	32 (28.3)	1.03 (0.57–1.87)	0.913
Number of comorbidities	2 (2–3)	2 (1–3)	0.108 (−0.048 to 0.254)	0.162

Data are presented as n (%) except for the number of comorbidities, which is given as median (interquartile range); the corresponding mean ± standard deviation is reported in Supplementary Table S2. For binary variables the effect measure is the odds ratio, expressed as female versus male, and groups were compared using the chi-squared test; for the number of comorbidities the effect measure is the rank-biserial correlation and the comparison used the Mann–Whitney U test. CI, confidence interval.

**Table 2:** Baseline demographic and clinical characteristics according to sex (n = 213).

Variable	Female (n = 100)	Male (n = 113)	Rank-Biserial Correlation (95% CI)	*p*
Age (years)	77.0 (68.5–81.0)	67.0 (57.0–75.0)	0.392 (0.245–0.542)	<0.001
Systolic blood pressure (mmHg)	120 (106–140)	125 (100–140)	−0.018 (−0.176 to 0.143)	0.823
Diastolic blood pressure (mmHg)	70 (60–75)	75 (60–80)	−0.227 (−0.381 to −0.077)	0.004
Oxygen saturation on room air (%)	96 (93–98)	96 (93–97)	0.075 (−0.082 to 0.226)	0.339
Body temperature (°C)	36.8 (36.5–37.3)	36.7 (36.4–37.4)	0.059 (−0.098 to 0.211)	0.455
Glasgow Coma Scale	15 (15–15)	15 (15–15)	0.001 (−0.114 to 0.119)	0.992
qSOFA score	0 (0–1)	0 (0–1)	−0.011 (−0.150 to 0.132)	0.885

Data are presented as median (interquartile range); the corresponding means ± standard deviations are reported in Supplementary Table S2. Groups were compared using the Mann–Whitney U test; the effect size is the rank-biserial correlation, for which positive values indicate higher values in female patients. Statistical significance was defined as *p* < 0.05. CI, confidence interval; qSOFA, quick Sequential Organ Failure Assessment.

**Table 3:** Treatment interventions administered in the Emergency Department according to sex.

Intervention	Female (n = 100)	Male (n = 113)	OR (95% CI)	*p*
Supplemental oxygen, n (%)	28 (28.0)	32 (28.3)	0.98 (0.54–1.79)	0.959
Vasopressor therapy, n (%)	15 (15.0)	26 (23.0)	0.59 (0.29–1.19)	0.139
Broad-spectrum antibiotics, n (%)	88 (88.0)	99 (87.6)	1.04 (0.46–2.36)	0.931

Data are presented as n (%). Odds ratios are expressed as female versus male. Groups were compared using the chi-squared test. CI, confidence interval; OR, odds ratio.

**Table 4:** Laboratory parameters at Emergency Department admission according to sex.

Parameter	Female (n = 100)	Male (n = 113)	Rank-Biserial Correlation (95% CI)	*p*
Haemoglobin (g/dL)	11.79 (9.53–13.13)	12.45 (10.19–14.79)	−0.227 (−0.373 to −0.076)	0.004
Leukocytes (×10^9^/L)	13.96 (9.50–19.26)	14.79 (9.49–19.64)	−0.037 (−0.191 to 0.119)	0.645
Neutrophils (×10^9^/L)	11.43 (7.28–15.49)	12.50 (8.04–17.04)	−0.072 (−0.226 to 0.083)	0.369
Lymphocytes (×10^9^/L)	0.86 (0.53–1.39)	0.99 (0.57–1.44)	−0.048 (−0.202 to 0.104)	0.546
Neutrophil-to-lymphocyte ratio	12.66 (6.08–21.34)	11.49 (7.17–21.40)	−0.014 (−0.177 to 0.155)	0.863
C-reactive protein (mg/L)	132.9 (73.6–262.6)	184.4 (81.1–256.7)	−0.101 (−0.256 to 0.057)	0.203
Procalcitonin (ng/mL)	2.12 (0.70–11.12)	1.73 (0.67–7.38)	0.050 (−0.108 to 0.210)	0.528

Data are presented as median (interquartile range); the corresponding means ± standard deviations are reported in Supplementary Table S3. Groups were compared using the Mann–Whitney U test; the effect size is the rank-biserial correlation, for which positive values indicate higher values in female patients. CI, confidence interval.

**Table 5:** Documented source of infection according to sex.

Source of Infection	Female (n = 100)	Male (n = 113)	OR (95% CI)	*p*
Respiratory, n (%)	22 (22.0)	36 (31.9)	0.60 (0.33–1.12)	0.107
Digestive, n (%)	22 (22.0)	25 (22.1)	0.99 (0.52–1.90)	0.983
Documented urinary source of infection, n (%)	51 (51.0)	39 (34.5)	1.97 (1.14–3.43)	0.015
Cutaneous/soft tissue, n (%)	5 (5.0)	11 (9.7)	0.49 (0.16–1.46)	0.191
Unknown, n (%)	17 (17.0)	23 (20.4)	0.80 (0.40–1.60)	0.532

Data are presented as n (%). Categories are not mutually exclusive; percentages therefore sum to more than 100%. Sources were assigned from the final Emergency Department assessment and were not systematically confirmed microbiologically; they are therefore reported as documented sources. Odds ratios are expressed as female versus male. Groups were compared using the chi-squared test. CI, confidence interval; OR, odds ratio.

**Table 6:** Clinical outcomes according to sex.

Outcome	Female (n = 100)	Male (n = 113)	OR (95% CI)	*p*
Direct ICU admission from the ED, n (%)	14 (14.0)	21 (18.6)	0.71 (0.34–1.49)	0.368
In-hospital mortality, n (%)	7 (7.0)	9 (8.0)	0.87 (0.31–2.43)	0.790

Data are presented as n (%). Odds ratios are expressed as female versus male. Groups were compared using the chi-squared test. Ward admission is the exact complement of direct ICU admission and is therefore not reported separately. Direct ICU admission denotes admission to the ICU directly from the ED; transfers to the ICU after initial ward admission are not included. CI, confidence interval; ED, Emergency Department; ICU, intensive care unit; OR, odds ratio.

**Table 7:** Sex-related differences in clinical characteristics before and after adjustment for age.

Characteristic	Unadjusted OR (95% CI)	Age-Adjusted OR (95% CI)	*p*	BH-Adjusted *p*
Cardiovascular disease	2.35 (1.33–4.17)	1.58 (0.83–3.02)	0.166	0.221
Renal disease	1.71 (0.99–2.94)	1.32 (0.74–2.36)	0.341	0.341
Documented urinary source of infection	1.97 (1.14–3.43)	2.14 (1.20–3.81)	0.010	0.039
Respiratory source of infection	0.60 (0.33–1.12)	0.53 (0.28–1.02)	0.059	0.118

Odds ratios are expressed as female versus male and were obtained from logistic regression models containing sex alone (unadjusted) or sex and age (age-adjusted). *p* refers to the age-adjusted model; BH-adjusted *p* is the Benjamini–Hochberg adjusted value across the four comparisons in this table. These were planned secondary analyses and were not part of the primary analysis. All four models were fitted in the complete cohort of 213 patients. BH, Benjamini–Hochberg; CI, confidence interval; OR, odds ratio.

**Table 8:** Multivariable logistic regression analysis of clinical outcomes.

Model and Variable	Adjusted OR (95% CI)	*p*	Events/n
Direct ICU admission from the ED (primary outcome)			35/213
Sex (female vs. male)	0.64 (0.29–1.39)	0.257	—
Age (per year)	1.00 (0.97–1.04)	0.765	—
Number of comorbidities	1.37 (1.02–1.86)	0.038	—
In-hospital mortality (secondary)			16/213
Sex (female vs. male)	0.80 (0.27–2.35)	0.690	—
Age (per year)	1.01 (0.97–1.05)	0.614	—

Odds ratios are expressed as female versus male. Direct ICU admission denotes admission to the ICU directly from the ED; transfers to the ICU after initial ward admission are not included. The model for direct ICU admission was adjusted for age and number of comorbidities; the model for in-hospital mortality was adjusted for age only, in view of the limited number of events, and was planned as a secondary analysis. Age is modelled per one-year increment and number of comorbidities per additional condition. These are adjusted associational estimates and do not identify a causal effect of sex. CI, confidence interval; ED, Emergency Department; ICU, intensive care unit; OR, odds ratio.

## Data Availability

The datasets are not publicly available, but de-identified data may be provided upon request from Larysa Balulescu.

## Supplementary Materials

The following supporting information can be downloaded at: https://www.mdpi.com/article/10.3390/medicina62091776/s1. Figure S1: Directed acyclic graph of the assumed relations among sex, age, comorbidity burden, the intermediate variables and the study outcomes; Table S1: Software used for the analysis, with version numbers; Table S2: Means ± standard deviations for the demographic and clinical variables in Table 2 and for the number of comorbidities in Table 1; Table S3: Means ± standard deviations for the laboratory parameters in Table 4; Table S4: Sensitivity of the estimate for female sex to the adjustment set; Table S5: Post hoc exploratory analysis of effect modification by age; Table S6: Shape of the association between comorbidity burden and direct ICU admission from the Emergency Department. Text S1: Post hoc exploratory analysis of effect modification by age.
