# Supplementary material for: Sex-Related Differences in Clinical Characteristics and Outcomes Among Patients Clinically Managed for Suspected Bacterial Infection in the Emergency Department
Source: Medicina (Kaunas). 2026 Sep 16;62(9):1776. doi: 10.3390/medicina62091776 (PMC13609446; doi:10.3390/medicina62091776)
Supplement: Supplementary file 1 [file medicina-62-01776-s001.zip › medicina-4554680-supplementary.pdf]

Supplementary Materials

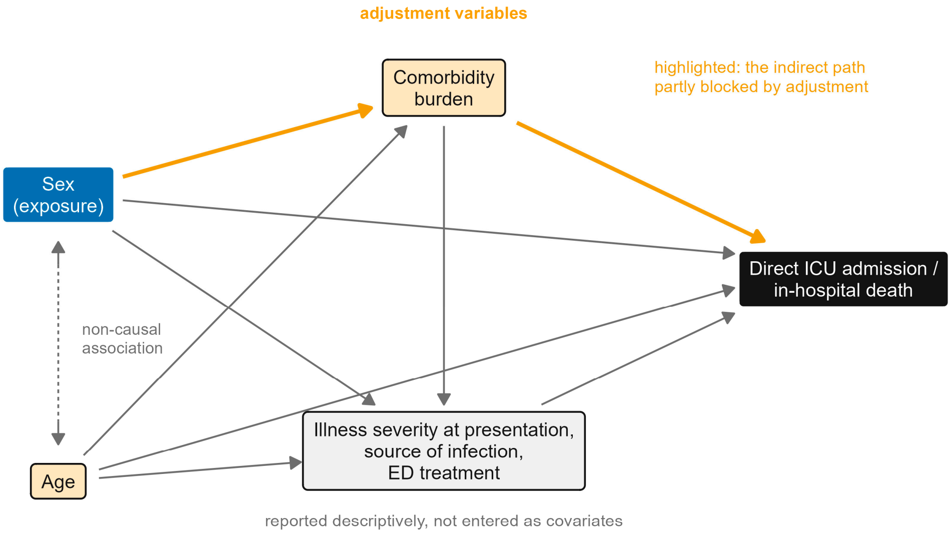

Figure S1. Directed acyclic graph of the assumed relations among sex, age, comorbidity burden, the intermediate variables and the study outcomes. Sex is the exposure. Age and comorbidity burden were entered as adjustment variables. Illness severity at presentation, source of infection and the treatment delivered in the Emergency Department lie between sex and outcome; conditioning on them would change the quantity being estimated, and they were therefore reported descriptively rather than entered as covariates. The highlighted path marks the indirect route from sex to outcome through comorbidity burden, which adjustment for comorbidity burden partly blocks; the unadjusted estimate is accordingly read as the total association and the adjusted estimate as the association remaining after adjustment. The dashed connector between sex and age denotes a non-causal association arising from differential survival to older age. The outcome node refers to direct admission to the intensive care unit from the Emergency Department. ED, Emergency Department; ICU, intensive care unit.

Table S1. Means ± standard deviations for the demographic and clinical variables presented in Table 1 and for the number of comorbidities presented in Table 2, according to sex. All continuous variables departed significantly from normality on the Shapiro–Wilk test; medians with interquartile ranges are therefore the primary presentation and are given in the main text. These summaries are reported to allow comparison with previously published cohorts.

**Variable**	**Female (n = 100)**	**Male (n = 113)**
Age (years)	72.1 ± 15.3	64.2 ± 13.7
Systolic blood pressure (mmHg)	123.3 ± 26.1	123.5 ± 27.0
Diastolic blood pressure (mmHg)	67.1 ± 12.5	72.3 ± 14.1
Oxygen saturation on room air (%)	94.6 ± 5.2	94.2 ± 4.6
Body temperature (°C)	37.1 ± 0.9	37.1 ± 1.0
Glasgow Coma Scale	13.9 ± 2.3	13.8 ± 2.3
qSOFA score	0.66 ± 0.78	0.72 ± 0.89
Number of comorbidities	2.5 ± 1.2	2.3 ± 1.3

Table S2. Means ± standard deviations for the laboratory parameters presented in Table 4, according to sex. Medians with interquartile ranges are given in the main text.

Variable	Female (n = 100)	Male (n = 113)
Haemoglobin (g/dL)	11.36 ± 2.40	12.52 ± 3.46
Leukocytes (×10^9/L)	16.26 ± 12.60	15.63 ± 8.53
Neutrophils (×10^9/L)	12.41 ± 7.53	13.29 ± 8.06
Lymphocytes (×10^9/L)	1.42 ± 2.11	1.16 ± 0.80
Neutrophil-to-lymphocyte ratio	14.84 ± 10.68	16.11 ± 13.47
C-reactive protein (mg/L)	169.2 ± 129.8	197.2 ± 147.4
Procalcitonin (ng/mL)	15.61 ± 41.00	12.35 ± 31.35

Table S3. Software used for the analysis, with version numbers.

Component	Version	Purpose
R	4.6.0	Base environment (released 2026-04-24)
stats	4.6.0	Logistic regression, Mann-Whitney U test, chi-squared test and Benjamini-Hochberg correction; the stratified percentile bootstrap uses base sample() with a fixed seed
readxl	1.5.0	Reading the source data file
ggplot2	4.0.3	Figures
patchwork	1.3.2	Figure composition
logistf	1.26.1	Firth penalized likelihood

Text S1. Post-hoc exploratory analysis of effect modification by age.

After the planned analyses had been completed, a sex-by-age interaction term was added to each of the two outcome models as a post-hoc check for effect modification. These analyses were not part of the analysis plan. They are reported for completeness, are hypothesis-generating, and carry no inferential weight.

For direct ICU admission from the Emergency Department, the interaction term was added to the primary model containing sex, age and comorbidity count. It was not significant on a likelihood ratio test ($p = 0.323$), and the model gives no indication that the association between sex and direct ICU admission varies with age.

For in-hospital mortality, the interaction term was added to the model containing sex and age, and reached nominal significance ($p = 0.024$). This result should not be read as evidence of effect modification. It rests on 16 events and three estimated parameters, which is far too few to support a reliable interaction test, and two further checks show the estimate to be unstable.

First, the effect of age on mortality was estimated separately within each sex. Neither sex-specific estimate was itself significant: the odds ratio per additional year was 1.09 (95% CI 0.99–1.21) in women and 0.98 (95% CI 0.93–1.02) in men. The apparent interaction therefore rests on a contrast between two estimates that are individually compatible with no age effect at all.

Second, the interaction test was repeated 16 times, each time omitting one of the 16 deaths. The p value ranged from 0.013 to 0.055 across these refits, and 1 omission raised it above the conventional 0.05 threshold. A finding that a single observation can overturn is not a stable one.

Taken together, these checks indicate that the nominally significant interaction for in-hospital mortality is a product of the small number of events rather than evidence that the association between sex and mortality depends on age. Determining whether such an interaction exists would require a cohort with substantially more outcome events.

Table S4. Post-hoc exploratory analysis of effect modification by age. These analyses were carried out after the planned analyses had been completed. They are hypothesis-generating, carry no inferential weight, and do not provide evidence of effect modification. The interaction for in-hospital mortality rests on 16 events and three estimated parameters, and was not robust to the omission of individual deaths.

Analysis	Estimate	p value	Note
Sex-by-age interaction, direct ICU admission	-	0.323	Model: sex + age + comorbidity count + sex:age; likelihood ratio test
Sex-by-age interaction, in-hospital mortality	-	0.024	Model: sex + age + sex:age; likelihood ratio test; 16 events, 3 parameters
Age effect on mortality, women	OR 1.09 (95% CI 0.99–1.21)	-	Per one-year increment, fitted within women only
Age effect on mortality, men	OR 0.98 (95% CI 0.93–1.02)	-	Per one-year increment, fitted within men only
Leave-one-out range of the interaction p value	0.013 to 0.055	-	Each of the 16 deaths omitted in turn; 1 omission raises p above 0.05

Table S5. Sensitivity of the estimate for female sex to the adjustment set, for the primary outcome of direct ICU admission from the Emergency Department. Odds ratios are expressed as female versus male. The estimate is essentially unchanged whether or not the comorbidity count is included, so the conclusion regarding sex does not depend on that adjustment decision. The p values are from Wald tests of the coefficient for sex; the unadjusted value therefore differs slightly from the chi-squared p value reported in Table 6 (0.368).

Model	Adjustment set	OR for female sex (95% CI)	p value
Unadjusted	None (total association)	0.71 (0.34–1.49)	0.369
Sensitivity model	Age only	0.63 (0.29–1.37)	0.248
Primary model (Table 8)	Age and comorbidity count	0.64 (0.29–1.39)	0.257

Table S6. Shape of the association between comorbidity burden and direct ICU admission from the Emergency Department. The primary model enters the comorbidity count linearly. A likelihood ratio test for departure from linearity, in which the count entered as four categories is added to the linear model, does not reject linearity (p = 0.178), but with 35 events it has little power to do so. The crude rates are step-like rather than graded: they rise from 5.4% among patients with none or one condition to 22.2% among those with two and do not increase further. The per-condition odds ratio reported in Table 8 should therefore be read as a summary of an overall trend rather than as the effect of each additional condition. Odds ratios are adjusted for sex and age, with 0–1 conditions as the reference category.

Number of comorbidities	Patients, n	Direct ICU admissions, n (%)	Adjusted OR (95% CI)	p value
0-1	56	3 (5.4)	1 (reference)	–

**Number of comorbidities**	**Patients, n**	**Direct ICU admissions, n (%)**	**Adjusted OR (95% CI)**	**p value**
2	63	14 (22.2)	5.20 (1.34–20.26)	0.017
3	52	8 (15.4)	3.49 (0.83–14.75)	0.089
4 or more	42	10 (23.8)	5.83 (1.37–24.81)	0.017
